# Forcing the Antitumor Effects of HSPs Using a Modulated Electric Field

**DOI:** 10.3390/cells11111838

**Published:** 2022-06-04

**Authors:** Carrie Anne Minnaar, Andras Szasz

**Affiliations:** 1 Wits Donald Gordon Academic Hospital, Johannesburg 2193, South Africa; carrie@onc-hyperthermia.co.za; 2 Department of Radiation Sciences, University of the Witwatersrand, Johannesburg 2000, South Africa; 3 Biotechnics Department, Hungarian University of Agriculture and Life Sciences, 2100 Godollo, Hungary

**Keywords:** oncology, hyperthermia, modulation, modulated electro-hyperthermia, bio-electromagnetics, transmembrane protein, raft, immunogenic cell-death, abscopal effect, nonthermal field effect

## Abstract

The role of Heat Shock Proteins (HSPs) is a “double-edged sword” with regards to tumors. The location and interactions of HSPs determine their pro- or antitumor activity. The present review includes an overview of the relevant functions of HSPs, which could improve their antitumor activity. Promoting the antitumor processes could assist in the local and systemic management of cancer. We explore the possibility of achieving this by manipulating the electromagnetic interactions within the tumor microenvironment. An appropriate electric field may select and affect the cancer cells using the electric heterogeneity of the tumor tissue. This review describes the method proposed to effect such changes: amplitude-modulated radiofrequency (amRF) applied with a 13.56 MHz carrier frequency. We summarize the preclinical investigations of the amRF on the HSPs in malignant cells. The preclinical studies show the promotion of the expression of HSP70 on the plasma membrane, participating in the immunogenic cell death (ICD) pathway. The sequence of guided molecular changes triggers innate and adaptive immune reactions. The amRF promotes the secretion of HSP70 also in the extracellular matrix. The extracellular HSP70 accompanied by free HMGB1 and membrane-expressed calreticulin (CRT) form damage-associated molecular patterns encouraging the dendritic cells’ maturing for antigen presentation. The process promotes CD8^+^ killer T-cells. Clinical results demonstrate the potential of this immune process to trigger a systemic effect. We conclude that the properly applied amRF promotes antitumor HSP activity, and in situ, it could support the tumor-specific immune effects produced locally but acting systemically for disseminated cells and metastatic lesions.

## 1. Heat Shock Proteins

The stress- or heat shock proteins (HSPs) represent a large family of highly conserved molecules vital in almost every living cell, on their surfaces, and in their extracellular microenvironments, throughout the cells’ lifetime regardless of their evolutionary level [1]. Their regulatory roles are complex and diverse, including protection from stresses, regulation of neurodevelopment [2], and modulating immune functions [2]. Some HSPs are present at relatively constant levels, while the large quantities of the expression of others appear only in response to stress [3]. Most HSPs act as molecular chaperones which play a role in protein maturation or degradation and can either reverse or inhibit the denaturation or unfolding of cellular proteins in response to stress. There are many different families of HSP chaperones. Each family acts differently to aid protein folding, as summarized by Horvath and colleagues in their review [4]. Additional functions include blocking aggregation, facilitating protein translocation through intracellular compartments, and the maintenance of steroid receptors and transcription factors [4].

Any change in the dynamic equilibrium of the cell’s life, such as environmental stresses [5], pathogenic processes [6], diseases [7], or even psychological stresses [8], activates the synthesis of HSPs. The genetic orchestrator of the HSPs is the master transcription factor, the heat shock factor (HSF) [9], which plays a role in disease and aging [10]. The HSF1 [11], and some other inducers, such as hypoxia-inducible factor 1 (HIF-1) [12], matrix metalloproteinase 3 (MMP-3), and heterochromatin protein 1 [13], trigger the production of the relevant HSPs in a coordinated manner.

The roles and impacts of HSPs are highly complex. The complexity determines their role in immune modulation and in cancer development. For example, increased expressions of the members of the HSP70 family HSPa1a, HSPa1b, and HSPa7 are associated with a poor prognosis in human colorectal cancer observed in the comparison of the pretreatment tumor samples with the clinical data. However, the increased expression of the same family member, HSPa9, in tumors was associated with a favorable prognosis [14]. Heat shock proteins also play a role in many human pathologies. Low levels of HSPs are typically seen in Type II Diabetes and neurodegenerative diseases, while malignant cells have abnormally high levels [15].

While the primary function of most HSPs is the protection of cells against stresses, their function is not limited to protection on a cellular basis. Under certain conditions, they participate in the collective protection of multicellular structures and tissues and may even play a role in systemic processes [16,17].

HSPs are able to transport intracellular antigenic peptides to antigen-presenting cells (APCs), which produce antigen-specific cytotoxic T-lymphocytes (${\mathrm{CD8}}^{+}$). During this process, the intracellular HSPs (iHSPs) transfer through the cellular membrane and into the ECM [18]. The secretion of HSPs on the membrane (mHSPs), or release of them into the ECM (eHSPs), appears to be associated with lysosomal endosomes or via the release of exosomes containing HSPs [19]. The iHSP may be translocated to the cellular membrane via the same transport pathway of secretory lysosomes during this process. A fusion between the lysosome and cell membrane occurs, followed by the insertion of the lysosomal membrane protein LAMP1 into the extracellular portion of the cell membrane and the secretion of the HSPs, along with other lysosomal proteins, into the ECM [20]. All three variants of HSPs concerning their position to the plasma membrane (iHSP, mHSP, eHSP) regulate the complex interactions of the cells with their environment but have different roles. Intracellular HSPs are typically cytoprotective, while membrane-associated and extracellular HSPs are not.

Furthermore, iHSPs have active essential functions in various locations within the cell, including the nucleus, the mitochondria, the endoplasmic reticulum (ER), and the cytosol [21]. The mHSPs and eHSPs have a physiological role in inflammation and pro-, and antitumor activity [22].

Intracellularly, the HSPs as molecular chaperones assist in maintaining the balance between the intracellular proteins, participate in the regulation of apoptosis, and protect the cells from stress [23] caused by various external stressors, such as hypoxia, thermal or oxidative stress, mechanical stressors, or even electromagnetic interactions. Membrane-bound HSPs modulate membrane characteristics such as fluidity, permeability, and secretary routes, while eHSPs are important mediators of intercellular signaling [4].

The different families of HSPs represent the further functional division at numerous locations. Therefore, each has specific activities and functions in a cooperative network of homeostatic control.

### HSPs: Friend or Foe in Oncology

The multifunctional behavior of HSPs results in opposing actions. They may either support cellular defense [1] or, in some instances, promote cellular death [24]. This dual function of HSP has led them be classified as a “friend” or “foe” [25,26,27]. The HSPs participate in the maintenance of the dynamic and complex homeostatic balance. The stochastic mechanisms of the HSPs’ balance their contribution to regulative processes, participating as promoters or suppressors. The decision between the two opposing behaviors depends on their microenvironment’s conditions and interactions. The balancing creates a “double-edged sword” [28,29] exhibiting both sides: inflammatory and anti-inflammatory; protumoral and antitumoral; immune-stimulatory and immune-suppressant, etc. In standard homeostatic conditions, HSPs support cell protection in the case of healthy functioning cells and support cellular death in the case of cellular dysfunction.

The distinction of functions in malignancy is rather complex. The individually well-functioning cells with unicellular preference renounce multicellularity, causing fundamental challenges in malignancy decisions [30]. The malignant cells vividly function, immortally proliferating as unicellular units, but their activity is destructive to the system, in part, where they are located. In this meaning, the malignant proliferation and the evolutional unicellular invasion have a lot in common [31]. The phenomenon is similar to atavism [30], considering the self-ruled unicellular activities. The loss of multicellular connections potentiates adaptability. These cells are robustly vivid. The malignant cells do not use the living advantages of collectivism; their individualism is predominant [32]. However, unicellular autonomy requires nutrition-rich environmental conditions for survival [33]. Cancer has a supportive environment provided by their healthy host. Cancer modifies its environmental conditions for support, and the homeostatic control tries to “heal” the abnormality [34]. The cancerous process avoids natural apoptosis [35]. The HSPs protect the malignant cells and appear as a “foe” of the organism in these processes. The curative task is straightforward: favor converting the role of HSPs to support cellular death.

Sensing the immunosurveillance of the system could serve as the reversing factor from a foe to a friend. The effects of the stress proteins were recognized early on in immunology [36] and have become a popular topic in the emerging field of immuno-oncology [37]. Furthermore, the use of HSPs as biomarkers of environmental analyses [38], prognoses [39], immune surveillance [12], and as therapeutic targets in malignant [40,41] and other diseases [42], is rapidly expanding.

Most oncological treatment methods cause extra stress, which induces HSPs. The increased HSP synthesis is seen after conventional hyperthermia [43], chemotherapy [44], radiotherapy [45], and even phototherapy [46]. The protection of the malignant cells depends on the chaperone functions of iHSPs from the HSP70, HSP27, and HSP90 families [13,47]. Overexpression of specific HSPs provides a selective advantage for malignant cells to inhibit apoptosis, promoting tumor metastasis and influencing immune responses for their benefits [48,49,50].

Stress also induces the translocation of iHSPs to the malignant cell’s membrane [51], forming mHSPs [23]. The mHSP is dual-action [52] as well, and could support [53] and suppress the survival abilities [54] of the malignant cells. The signaling of mHSPs may subsequently alert the NK cells to the presence of the malignant cells [55] as the first sign of immune activation. The extracellularly released HSPs could play a crucial role in tumor immunity [56]. In addition to the iHSP→mHSP→eHSP conversion sequences, the iHSP may be liberated directly into ECM from necrotic cells, providing eHSP without intermediate location on the plasma membrane.

## 2. Electromagnetic Method of Hyperthermia in Oncology

Electromagnetism is one of the active factors in biological processes, and it has a broad therapeutic application in oncology. One of the widely used treatments is the local-regional heating of the tumor and its environment. The absorption of electromagnetic energy heats the tissues in these therapies utilizing radiofrequency (RF) and microwaves [57,58]. It is a complementary oncological treatment applied in combination with the relevant standard protocols to support and enhance the effects of various other oncotherapies.

The heat stress-induced iHSPs present a significant challenge for oncological hyperthermia treatment because these chaperones’ elevated level develops treatment resistance and promotes the malignant processes [59]. The HSP27, HSP70, and HSP90 chaperone families reduce the tumor-suppression ability to support angiogenesis and metastases [59,60]. The heat shock regulator HSF1 plays a considerable role in tumorigenesis, thus, its knockdown significantly reduces the proliferation of cancer cells [61]. Consequently, developments of HSP inhibitors became a target of tumor research [62]. The disruption of HSP47 shows substantial sensitizing of such chemoresistant cancer as pancreatic ductal adenocarcinoma [63]. The inhibition of lactate dehydrogenase impairs the stress response and increases the radiosensitivity of many aggressive, otherwise radioresistant, tumors [64].

Paradoxically, hyperthermia has shown profound success in inhibiting cancerous growth. Hyperthermia combined with conventional chemo- and radiotherapies, surgery, and the emerging immunotherapies [65] achieved significant tumor destruction in clinical practices. It is an effective radio- and chemosensitizer and cooperates well with the immune system [66]. Multiple high evidence levels of the clinical studies prove the method’s success [67]. The strengths, weaknesses, and opportunities of hyperthermia applications in oncology have been analyzed in detail, showing general overall promise for exceptional success in treating tumors [68].

The apparent controversy between supporting or inhibiting tumor growth indicates, again, the complex behavior of heating interventions, which appears in the “double-edged sword” phenomena Cooperation with the natural homeostatic regulations could be a decisional factor to push the balance to the favorite side. One of the overall regulators of the homeostatic control is the immune-surveillance, which may have an excellent partner to win [69].

The bioelectromagnetic interactions potentially manipulate the locations and functions of the HSPs, driving the complex challenge of cooperating with the immune effects and re-establishing the healthy homeostatic balance.

Bioelectromagnetic energy absorption heats the targets. Heating has two fundamental concepts in oncological practice:

- One way is to heat the whole tumor volume isothermally. The applied focusing techniques intend to maximize the temperature of the tumor volume and minimize it in healthy surroundings [70]. The original heating goal is necrosis. The applied dose compares the actual cellular distortion to the necrosis achieved in vitro at 43 °C [71];
- Another way heats small selected targets, either artificially or naturally available centers in the tumor volume:
  – The method injects artificial (mostly inorganic metallic nanoparticles) into the tumors. These invasively placed exclusive energy absorbers [72,73] distribute locally throughout the tumor, absorb the energy selectively, and transfer heat to their environment;
  – The tumor’s heterogenic structure offers natural targets. The optimally chosen electromagnetic properties such as the frequency, the intensity, the phase, and the delivered time–information (modulation) allow for tuning to select the chosen particle (molecular cluster). For example, the membrane rafts (lipid micro-domains found in the plasma membranes of cells [74]) offer the perfect opportunity. The high electromagnetic contrast allows selection between the lipid-supported transmembrane proteins (membrane rafts) and their pure double lipid holding membrane material.

All heating methods induce the expression of various HSPs [75]. While homogeneous heating and the heating of artificially injected particles act only with their thermal activity, the natural particles may have additional nonthermal excitation by the field [76,77]. Note that an effect is considered nonthermal “when, under the influence of a field, the system changes its properties in a way that cannot be achieved by heating” [78].

The lipid raft micro-domains respond to electromagnetic fields [79]. Membrane rafts are highly heterogeneous and dynamic sterol- and sphingolipid-enriched domains, which may also involve protein interactions and compartmentalize cellular processes [80]. The rafts operate as a trigger of the intracellular processes [4]. The rafts collect dynamic proteins [74], including proteins with high lateral mobility in the membrane [81]. The raft’s size varies within the nano range, depending on the protein content in the cluster. The membrane of the malignant cells presents a significantly higher number of transmembrane proteins and their clusters than their nontumorigenic counterparts [82].

The heating of the molecular clusters on the tumor cells causes extreme stress on the cells, which can trigger programmed cell death [83]. The electromagnetic field extends the production of active HSPs [84,85]. The absorbed energy results in the heating of the target and resonantly excites the molecules, driving the signaling and the development of HSPs. A great part of the energy absorbed by the natural heterogeneities is nonthermal [86], and if characteristic, the optimally tuned electromagnetic wave could deliver energy for molecular excitations. The excitations focus on signal triggering and transmission and are involved in the various ionic and molecular interactions, focused on re-establishing the missing apoptosis in malignant cells. The process results in a subtle heterogeneously distributed thermal effect with the resonant conditions [87,88]. Research shows that the nonthermal resonant absorption adds to HSP expression [89] and function [90].

This review focuses on hyperthermia driving HSP activity in a “friendly way”. Our goal is to convert the HSPs’ role from “foe” to “friend”, promoting ICD of the tumor cells. This could shift the approach to treating malignancy with hyperthermia from targeting local disease to producing systemic activity.

**Figure 1:**
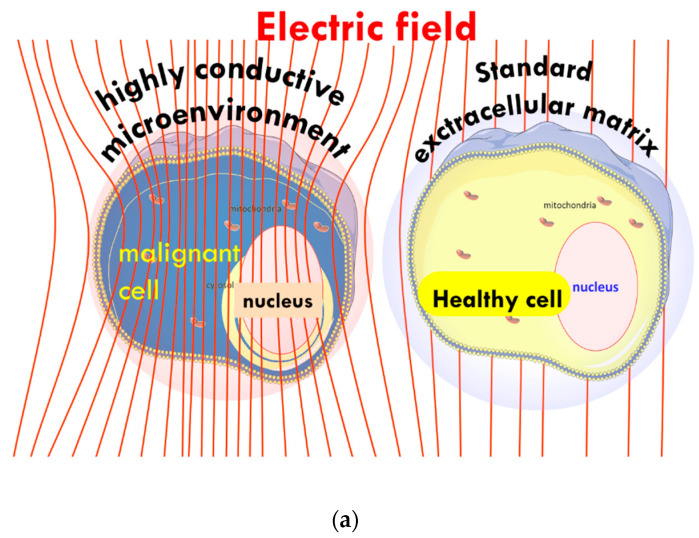

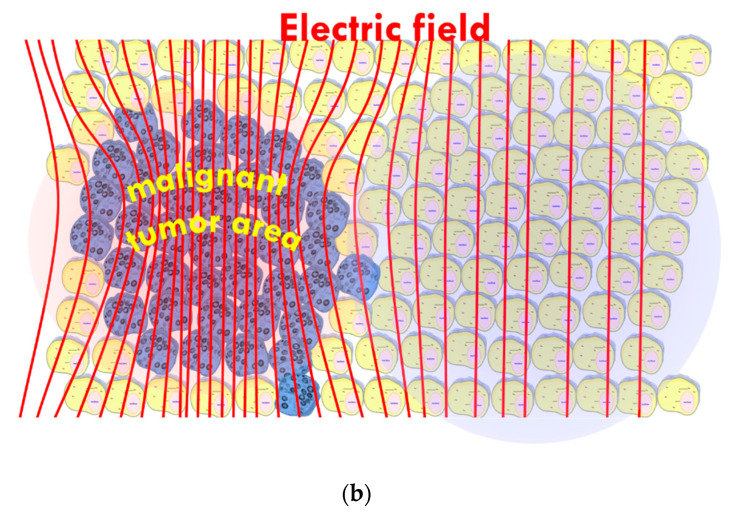
Schematic illustration of the current focus using impedance selection: (**a**) the microscopic illusion of an increase in absorbed energy by the malignant cell compared to the healthy cell, and (**b**) macroscopic illustration of the energy flowing more easily through malignant versus healthy tissue.

The heterogenic selection principle proposes a solution to the challenge of tissue selection in hyperthermia [91] by applying non-isothermal heating [92]. We use the principles of modulated electro-hyperthermia (mEHT), which has already demonstrated its clinical benefits, improving outcomes for patients when combined with chemotherapy and radiotherapy [93,94,95]. The mEHT synergically utilizes mild heating, which improves blood flow and perfusion [96] and the modulated electric field [97]. The heterogenic characteristics guide the mEHT-induced electric current through the target tissue. The unique attributes of malignant cells, such as their high proliferation rates, produce enhanced concentrations of ions in the ECM, resulting in their particular electromagnetic properties. As a result, the tumor microenvironment (TME) is more conductive and drives the flow of current through the target tumor using an appropriate frequency and modulation [87], Figure 1a,b.

**Figure 2:**
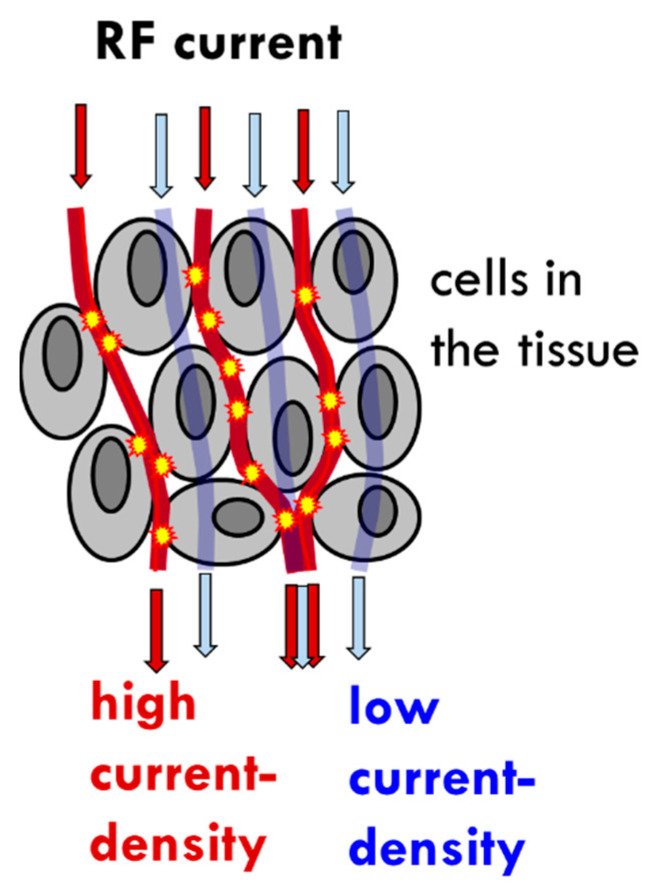
Nano-heating: schematic representation of the flow of current through malignant tissue, between the cells, and the absorption of the energy in the membrane rafts, leading to areas of high current density.

The selection uses the relatively high number of membrane rafts on the surface of malignant cells [82], which have a selectively high energy absorption rate of RF current [98] and appear to be the highest energy absorbers in the malignant tissue [98] (Figure 2).

**Figure 3:**
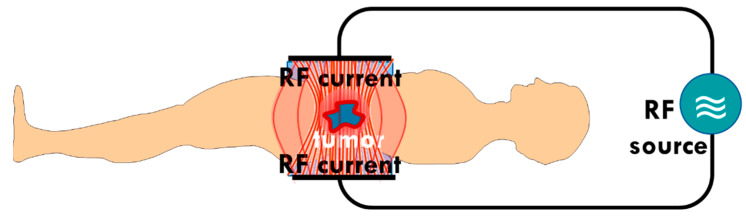
Schematic representation of the patient forming part of the tuned electric circuit. The low impedance of the tumor drives the current to select the malignant cells. There is precise, real-time control of the impedance matching of the patient and the tumor in situ.

The mEHT technology applies capacitive-coupled energy, using precise impedance matching [99] in order to create an electric field with characteristics that ensure the appropriate current density (j). The j acts thermally (Joule heat) and nonthermally (molecular excitation). The power of the thermal energy production depends on the $j^{2}$, while the j excites the molecules linearly. The j has to over-dominate the $j^{2}$ otherwise the electrical selection becomes negligible to the increased heating. A low current density (low power) must be applied to prevent the over-powering heat and ensure electrical selection. The mEHT uses a much lower power output than conventional hyperthermia [97]. This solution allows for the real-time control and adaption of the treatment to the patient, as the treated tumor forms a part of the tuned electric circuit [100] (Figure 3).

## 3. The Effect of mEHT on HSPs

The mEHT treatment induces up-regulation and increased expression of all major HSPs at an mRNA [101] and protein level [102] in HT29 xenografts. These preclinical studies have demonstrated the effects of mEHT on the up- and down-regulation of specific genes [83].

The heat-map of gene expression showed that mEHT induced significant up- or down-regulation of 48 transcripts of 39 genes compared to controls in the study on HT29 xenografts. The relative mRNA expression of HSP70 reached maximum expression four hours post-treatment [101]. Gene coding for members of the HSP70 family were up-regulated, including *HSPa1a*, *HSPa1b*, *HSPa4*, *HSPa6*, and *HSPa8*, and their co-chaperones *HSP40* (*DNAJB1* and *DNAJB4*) and Bag3. The transcription of *HSP90* alpha (*HSP90AA1*) and *HSP60* (*HSPD1*) was also up-regulated in HT29 xenografts. An increase in a broad spectrum of HSP families at an mRNA level was observed in HT29 xenografts four hours post-treatment [103].

The up-regulation of several genes, including *HSPb1*, *HSPa1a*, *HSPa1b*, and *HSPh1*, was also seen in triple-negative 4T1 breast cancer isografts treated with mEHT. This observation was noted 24 h post-treatment and was associated with inhibiting tumor growth and proliferation. *HSPa1a* and *HSPa1b* are the most common isoforms of the HSP70 family. The RNA sequencing showed significant *HSP70-1* (*HSP72*), *HSP70-2*, *HSPB1*, and chaperone HSP105 development 24 h after treatment, along with the up-regulation of the associated genes [104]. An increase in the *HSP70* concentrations around the peripheral margin of the tumors 24 h post-treatment was observed.

**Figure 4:**
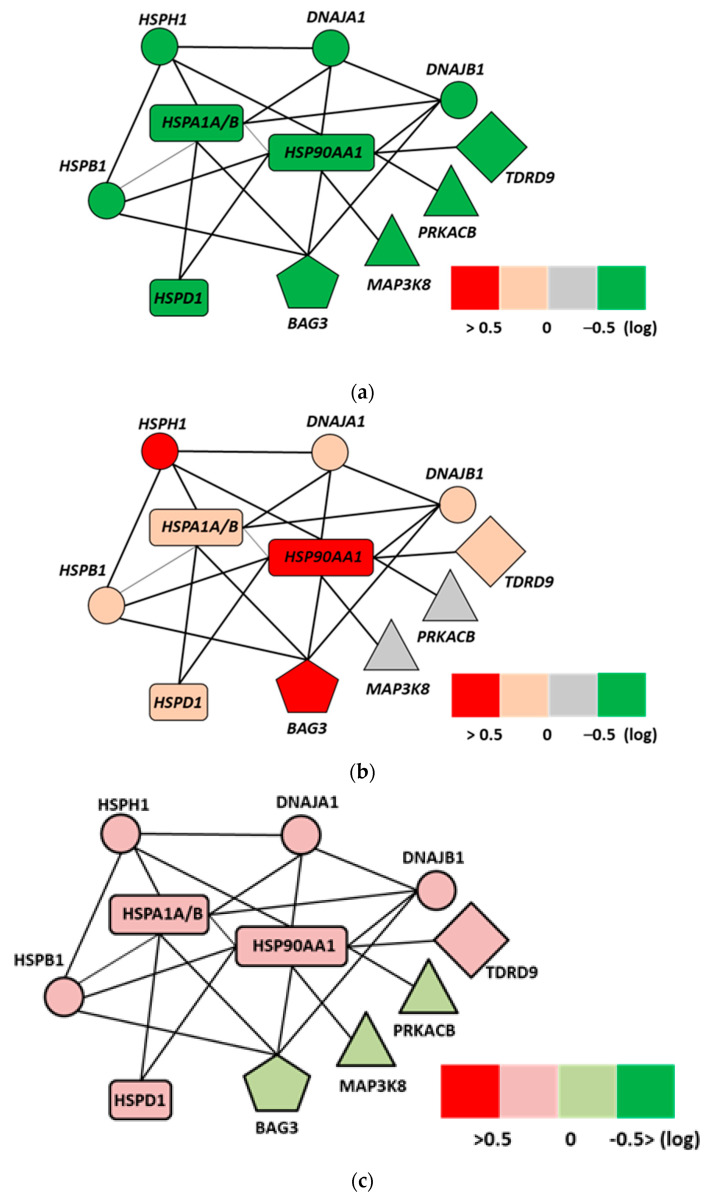
Ingenuity analysis of the network of HSP-related interactions at a genetic level [105]: (**a**) control samples (38 °C), (**b**) wHT-treated samples (42 °C), (**c**) mEHT (42 °C). Important differences between wHT and mEHT treatments at the same temperature: the less intensive tumor-protective HSP functions in the mEHT sample; the pro-tumor BAG3 remains nonregulated in the mEHT sample.

A gene chip analysis of the U937 (human lymphoma) cell line showed an activation of the cytoprotective gene network in samples heated with water-bath homogeneous heating (wHT). The up-regulation of genes, such as *HSP105* and *HSP90A*, have been shown to block apoptosis by interfering with caspase activation and directly and indirectly inhibit apoptosis. In this study, the HSPs appeared to play an antiapoptotic role as they prevented apoptotic cell death in the cells heated with wHT, but not in the cells heated with mEHT [105]. Here the gene map showed a distinct difference in the gene regulations between the water-bath and mEHT-treated samples at the same temperature. The ingenuity pathway analysis revealed the cell death’s specific gene network, including *EGR1*, *JUN*, and *CDKN1A* genes. Despite the same thermal condition, the mEHT treatment does not appear to activate the cytoprotective network while the water-bath treatment activates it. The activation of the ERK-JUN pathway was present only in the mEHT-treated samples. The FAS, c-JUN N-terminal kinases (JNK), and ERK signaling pathways dominate the apoptotic pathway. The ingenuity pathway study uncovered the HSP network (Figure 4a–c), which significantly differs from the wHT at the same temperature. As the temperatures in both heating methods were the same, the difference in pathway activations is most likely due to the electric field effects [105].

**Figure 5:**
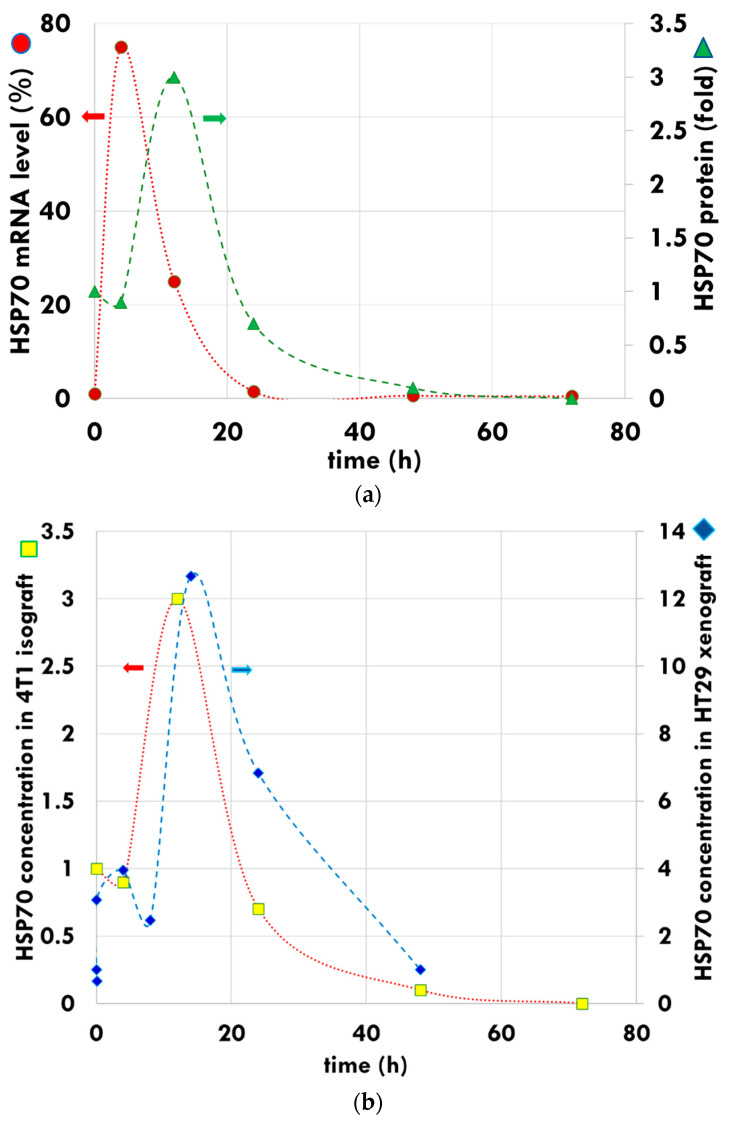
Development of iHSP by mEHT. (**a**) The HSP70 peak is indicated at mRNA level (dots, left axis) approximately eight hours earlier than the measured HSP70 folded protein peak (triangles, right axis). (**b**) The HSP peak has similar features in the measurements of the 4T1 isograft (square, left axis) and HT29 xenograft (diamond, right axis). The curves are for guiding the eye.

The level of mRNA-associated iHSP70 peaks at four hours post-treatment in 4T1 isograft models [104], while the HSP protein-associated expression reaches its maximum between 12 and14 h (Figure 5a), and decreases to the baseline concentration after 48 h in the 4T1 isograft [106] and the HT29 xenograft [101] samples (Figure 5b).

**Figure 6:**
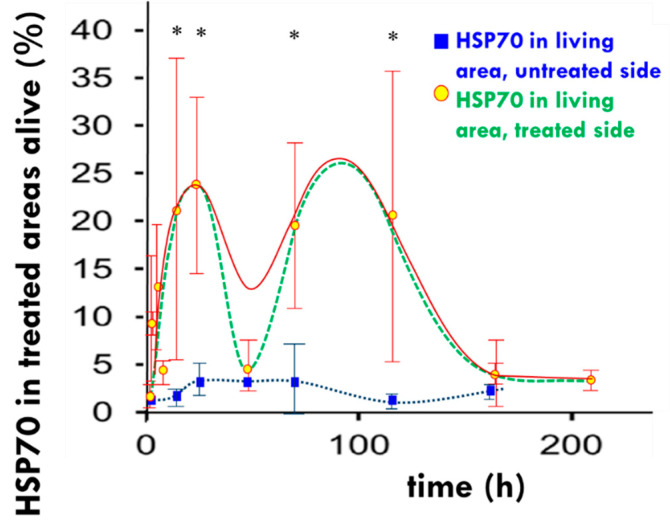
The measured development of HSP70 (dots, solid line). The dual peak pattern represents two types of HSP70. The first is primarily anti-apoptotic connected to the chaperone functions, while the second is pro-apoptotic as a part of the immunogenic cell death. The dashed line approximately represents the two peak separations. The dotted line shows the development of HSP70 in the untreated tumor of the immunocompromised murine HT29 xenograft model. The asterisks on the top indicate significant differences between the HSP70 recorded on the treated side and the untreated side. The curves are for guiding the eye. * *p* < 0.05.

It is clearly shown that the up-regulation of the HSP70 in the HT29 xenograft model treated with mEHT increases, peaking at 14 h post-treatment, followed by a decline and a return to baseline levels at 48 h. The distribution of HSP70 shows a large increase in iHSPs during this first peak. The second peak in HSP expression occurs between 72 and 120 h post-treatment, followed by a return to baseline levels after 168 to 216 h post-treatment (Figure 6). The second peak is likely a result of the release of mHSPs into the extracellular environment producing eHSPs [101].

**Figure 7:**
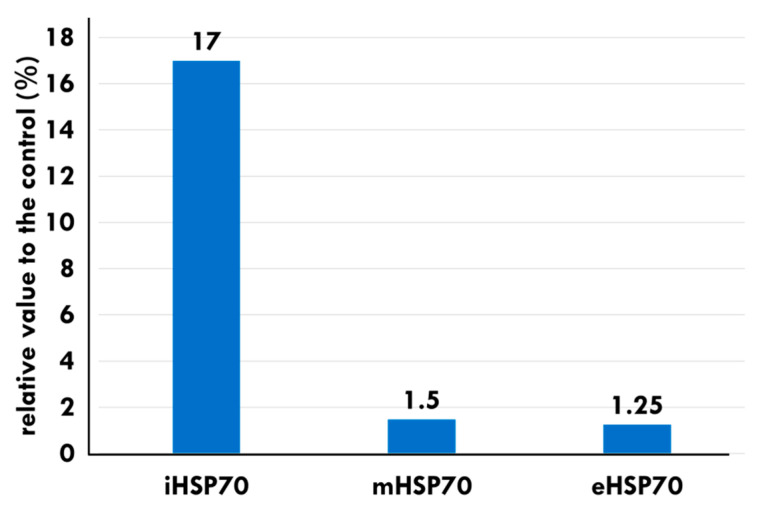
The localization distribution of HSP70s in B16F10 melanoma allografts at 24 h [107]. Massive apoptosis appeared despite increased chaperoning by iHSP70.

The levels of mHSP70 and eHSP70 also show an increase in B16F10 melanoma allograft models treated with mEHT, compared to the control, at 24 h post-treatment (Figure 7).

**Figure 8:**
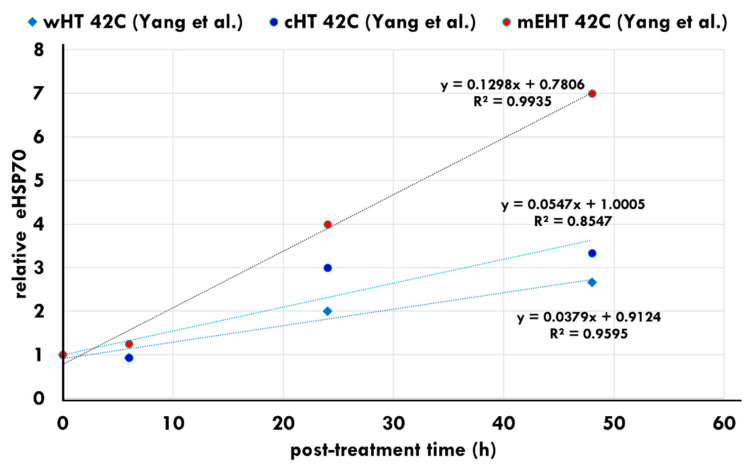
eHSP concentrations following water heating (wHT), capacitive heating (cHT), and heating using mEHT. The increase in eHSPs is significantly higher following mEHT heating compared to the other heating techniques [75].

Yang et al. showed that heat stress in the HepG2 cell line produces massive increases in iHSPs following all heating methods. The secretion of eHSP70 also appears post-treatment between 24 and 48 h in all heating methods. However, the levels of eHSPs are significantly higher following mEHT treatments [75] (Figure 8). The data of 24 h post-treatment show an accelerated increase in the eHSP concentration. The increase in the eHSP70 expression was also noted in vivo in B16F10 melanoma allografts [107] and in HT29 colorectal xenografts [105].

**Figure 9:**
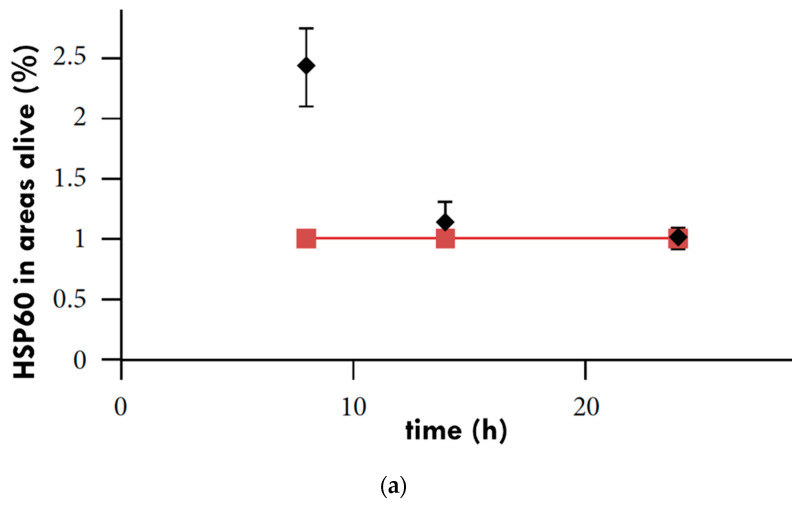

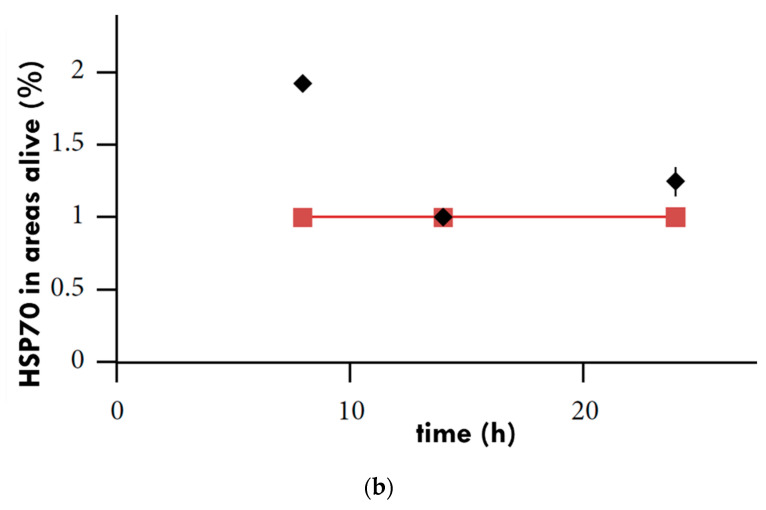
Relative protein expression of (**a**) HSP60 and (**b**) HSP90 in murine models. The diamonds represent HSP levels in treated tumors and the squares represent untreated tumors in the same mouse [102].

The inoculation of tumors into two different regions in vivo, e.g., the left and right femoral region [108], or the femoral and thoracic region [109], is a popular method for evaluating tumor responses to treatment as each tumor has its control in the same animal. When both mice’s femoral regions were inoculated with xenografted human colorectal cell line (HT29) tumors [108], the right tumor of each mouse had a treatment with 30 min of mEHT, and their left tumor remained untreated. After treatment, resected samples showed an increase in HSP60 and HSP70 concentration in the treated tumors, peaking at eight hours post-treatment [102] (Figure 9a,b).

**Figure 10:**
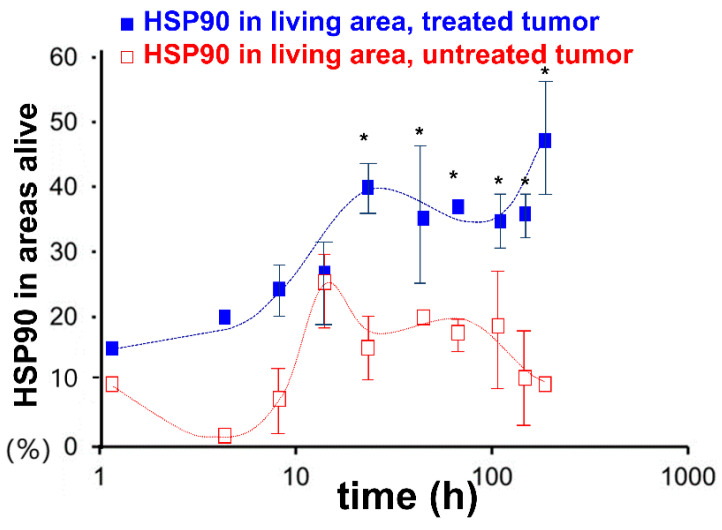
Graph showing the significant increase in HSP90 in the relative mask area (rMA—calculated by dividing the stained area by the whole annotation area), seen between 24 and 216 h posttreatment in the treated tumor cells (solid square markers) compared to the untreated tumor cells (empty square markers) in the morphologically intact areas from excised murine-model tumors (* *p* < 0.05) [101].

In a follow-up study by Andocs et al., mice were inoculated with HT29 xenograft cells in both hind legs. One of the resulting tumors in each model was treated with mEHT and the other was left untreated. A consistent increase in HSP90 was observed in the tumors treated with mEHT. In these tumors, two peaks in the expression of iHSP90 were observed; the first at ~24 h post-treatment and the second between 168 and 216 h post-treatment [101] (Figure 10). An increase in HSP90 expression was also observed in the untreated tumor in each model [101]. In the untreated tumor, the HSP90 expression peaked at ~14 h post-treatment, reaching levels close to those seen in the treated tumor at ~14 h post-treatment, before declining. The observation that the untreated tumor also responds with an increase in the level of HSP90 may suggest the presence of cross-talk between the two tumors, although this is speculative and further investigation is needed to confirm this.

## 4. The Effect of mEHT on Apoptosis

The results of the preclinical apoptosis studies suggest that despite the increased expression of iHSPs, the mEHT has the potential to inhibit tumor growth and support the development of an apoptotic process [110]. When HSP70s reach a peak at approximately 12 h post-treatment, the complex stress on the cells exhausts the HSP response and the subsequent protection capability of the HSPs at 24 h in 4T1 isografts [106].

In the event that the protective mechanisms from the iHSPs are unable to restore normal cell functions after exposure to stress, the stress on the cell results in cell cycle arrest, which is typically by caspase-dependent apoptosis [106]. During caspase-dependent apoptosis, caspase-3 is activated, forming cleaved caspase-3 (cCas3). To understand HSPs and their role post-induction from mEHT treatments, several markers, including cCas-3, have been studied alongside HSPs. The expression of iHSPs were found to peak at approximately 4 h post-treatment with mEHT and returned to normal levels at approximately 24 h post-treatment with mEHT [101,106].

**Figure 11:**
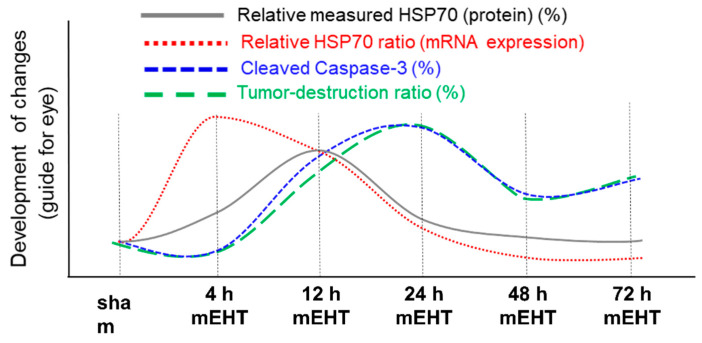
The trend of the development of tumor destruction, HSP expression, and cleaved caspase-3 at discrete time-points in 4T1 murine tumor isograft. The x-axis contains discrete steps, the curves are only to guide the eye.

The tumor destruction ratio (TDR,%) also peaked at approximately 24 h after the final mEHT treatment, suggesting that the protective mechanisms from the HSPs had been exhausted by this time. Furthermore, the proportion of the cCas3-positive regions overlapped extensively with the damaged regions of the tumor seen on consecutive sampling [106]. The progress of the development of HSP70 and the tumor destruction ratio alongside cCas3 shows well how the tumor-degradation overthrew the HSP70 protection (Figure 11). The timing suggests that the cCas3-dependent apoptosis plays a major role in destroying the tumor post-mEHT treatment in the 4T1 murine tumor isograft.

Apoptosis-inducing factor (AIF), a flavoprotein that resides in the mitochondrial intermembrane space, may also have a role in apoptosis caused by mEHT [111]. Following cellular stress, AIF translocates to the nucleus and triggers chromatin condensation and large-scale DNA degradation, inducing caspase-independent apoptosis [112]. AIF expression provides an additional signal pathway for apoptosis [110,111]. This process has the potential to affect untreated tumors as well [108].

**Figure 12:**
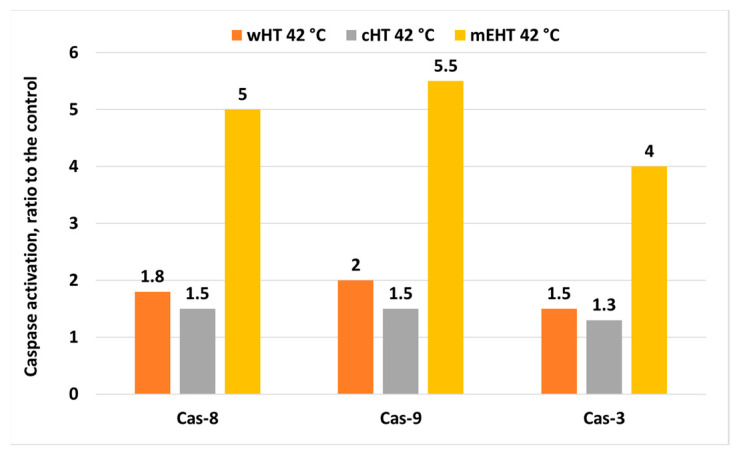
Apoptosis markers: cCas-3, -8, and -9, are increased in samples treated with mEHT compared with samples treated with water heating (wHT) and capacitive heating (cHT) [75].

The mEHT may induce other apoptotic pathways detected in vitro [75] (Figure 12). Various studies have shown that the complex apoptotic pathways triggered by mEHT result in higher rates of programmed cell death after cHT and wHT under the same thermal conditions (42 °C, 30 min) [75,113]. The multi-path apoptotic processes ensure massive apoptosis despite the various protective mechanisms of HSPs and XIAP.

The comparison of the wHT and mEHT under the same thermal conditions also shows significant differences in the intercellular calcium ($i{\mathrm{Ca}}^{2+}$) concentration [77,105,114]. An overload of ${\mathrm{Ca}}^{2+}$ intracellularly is detrimental to the health of the cell as it may increase their susceptibility to apoptotic cell death. The ${\mathrm{Ca}}^{2+}$ overload also causes the apoptitic destruction of tumor cells following the application of a modulated field [114]. Furthermore, mEHT treatment triggers the up-regulation of the E2F1 protein, which is involved in E2F1-mediated apoptosis [83] in various glioma cell lines. Apoptosis facilitators (such as PUMA and $p21^{\mathrm{waf}1}$ (also known as wild-type p53-activated fragment 1: WAF1) [115]), increased rapidly following the preclinical treatment of melanoma with mEHT and a significant reduction in tumor size was observed following a stress response signaled by iHSP70 and mHSP70 [107].

Another preclinical study on a melanoma model treated with mEHT [116] showed NK-cell infiltration into the tumor. The mHSP facilitates the NK-infiltration [117], which can explain the role of mEHT.

**Figure 13:**
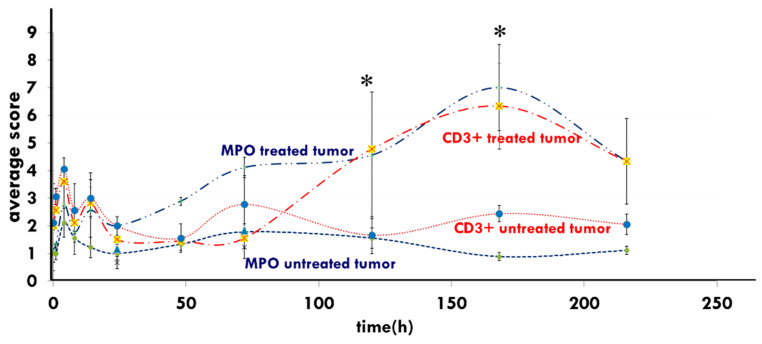
Induced immune reactions appear in both the Myeloperoxidase (MPO) and the $\mathrm{CD}3^{+}$-T–cells’ peak at one-week post-treatment in the HT29 xenograft [103]. (* means that *p* < 0.05).

Myeloperoxidase (MPO, peroxidase enzyme) positive neutrophil granulocytes (and monocytes) were significantly elevated in xenografts treated with mEHT compared to control tumors from 48 h to approximately 216 h post-treatment [103] in HT29 xenograft studies. The enrichment of MPO may further enhance cell destruction in mEHT-treated samples, which has support from the peak of the CD3+ T-cells’ density at the same peak as MPO at 168 h (Figure 13).

**Figure 14:**
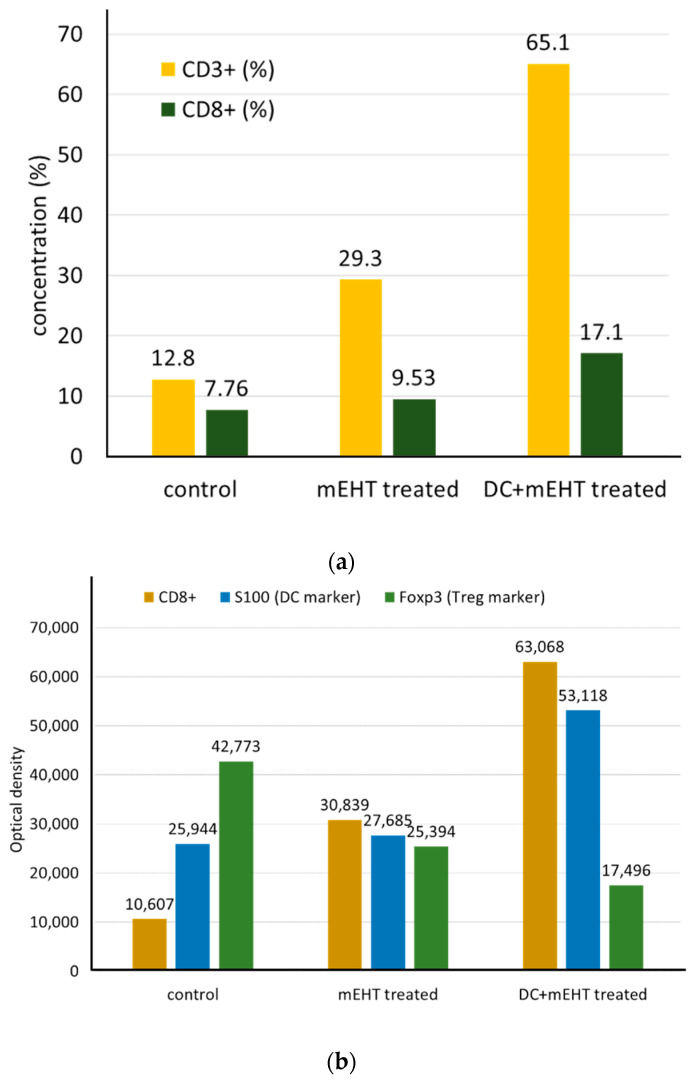
The mEHT treatment produced increased $\mathrm{CD}3^{+}$ and $\mathrm{CD}8^{+}$ cells, and the addition of immanentizing DC therapy enhanced this. (**a**) The distribution of $\mathrm{CD}3^{+}$ and $\mathrm{CD}8^{+}$ T-cells shows increased concentration after mEHT. (**b**) The optical density measurements of S100 DC and Foxp3 $T_{\mathrm{reg}}$ markers show the increased antitumor immune activity of mEHT and the addition of DC enhances the effects.

Modulated electro-hyperthermia may improve the immunological tumor microenvironment, followed by dendritic cell (DC) immune support [113]. This additional DC therapy is significant for advanced cases accompanied by worsening immune surveillance. The genetic information release delivered by eHSPs results in antigen-presenting and consequently elevated levels of cytotoxic T cells in the region. The increased activity of the $\mathrm{CD}8^{+}$ cytotoxic T-cells by mEHT treatment appeared in a squamous cell carcinoma SCCVII allograft after adding DCs to boost immune activity [109] (Figure 14a,b).

**Figure 15:**
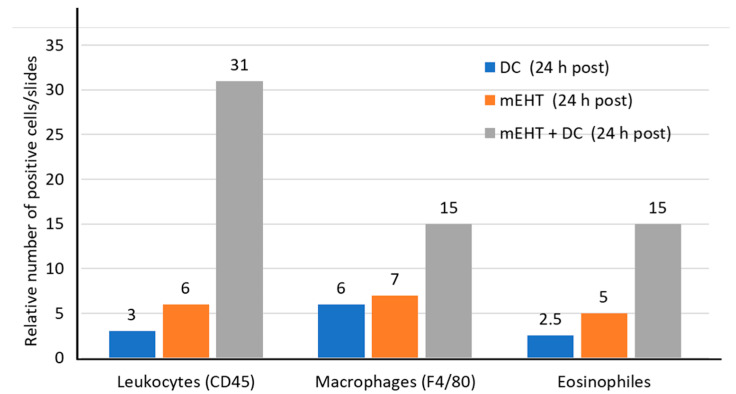
Increased concentration of leukocytes, macrophages, and eosinophils seen after the administration of mEHT combined with DC therapy.

The additional DCs appear to boost the leukocyte invasion of the tumor and support the macrophages and eosinophils (the T-cell organizers) in the CT26 allografts [113] (Figure 15).

**Figure 16:**
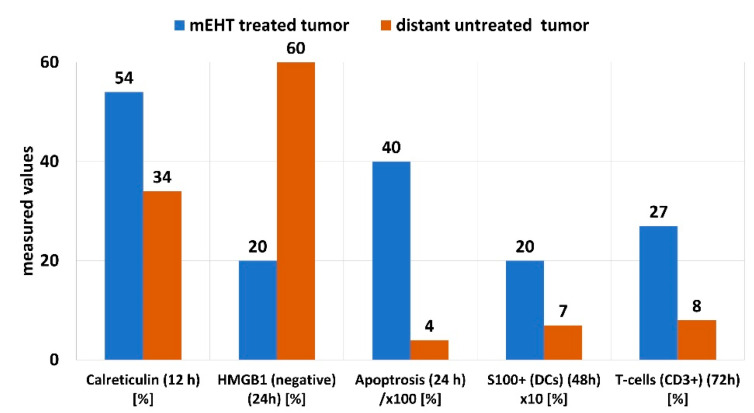
DAMP-related activity in C26 allografts following treatment with mEHT.

The eHSP70 appears together with a set of other molecules, forming a damage-associated molecular pattern (DAMP), which includes the release of calreticulin (CRT), high-mobility group Box 1 (HMGB1), and adenosine triphosphate (ATP). The cytoplasmic CRT translocates to the plasma membrane during the early stages (four hours) after treatment with mEHT in HT29 xenografts [103]. The DAMP activity measured in C26 allograft is illustrated in Figure 16 [108].

The addition of other stimuli supporting mEHT has enhanced the success of tumor-specific immune activation. The combination with fluorescently labeled primary NK cells (NK92MI) increased the NK cell penetration to the tumor [116], and the herbal immune-stimulator marsdenia tenacissima (MTE) enhanced the DAMP and the DC maturation by genetic information; delivered by eHSP70 [116].

## 5. Clinical Evidence of a Systemic Immune Effect Induced by mEHT

The radiation-induced abscopal effect does not frequently appear in clinical practice. Radiation therapy demonstrated the first observable abscopal effect [118] as a less common systemic response to ionizing radiation in which non-irradiated lesions respond after irradiation of the primary treatment site [119,120]. Unlike the bystander effect, the abscopal effect describes the response of untreated distant metastases to the local treatment of the primary tumor [121]. Activating tumor-specific immune mechanisms is responsible for the abscopal effect [120,122,123]. Only a few case studies deal with the abscopal phenomenon yearly [122,123]. However, the mEHT actively produces clinically observed abscopal phenomena. Following the preclinical studies of abscopal effects [108,109,124,125], case reports [126,127,128,129] demonstrated the clinical applicability. Some clinical studies validated the effects for various tumor entities [130,131,132,133]. Clinical trials for brain tumors show the combination of mEHT with viral immuno-boosting. The treatments showed significant improvements [134,135,136,137,138]. The combination with viral immune support also works for ovarian cancers [139].

Minnaar et al. (2019), in a Phase III mEHT study, described the possible observation of the abscopal effect in 24% of women with locally advanced cervical cancer and extra-pelvic nodal disease [140]. Cisplatin (either one or two doses) was also administered to some patients considering their renal function and hematological toxicity. In the subgroup, the frequency of the visualized abscopal effect (evaluated by 18F-FDG PET/CT scans pre-treatment and at six months post-treatment) was not associated with chemotherapy administration. The administration of mEHT significantly predicted the likelihood of complete metabolic resolution of all pelvic and extra-pelvic malignancies.

## 6. Discussion

The high biological heterogeneity of the tumor, and its structural and functional differences from the healthy host tissue, are apparent from the electromagnetic and thermal variability of the energy transmission and absorption [141]. These differences allow the targeting of malignant tissue over healthy tissue, using energy deposition and heat. When applied correctly, the energy deposition stimulates the production of HSPs in malignant tissue to act in favor of the host organism, supporting the immunogenic-related cell death of the malignant cells, potentially promoting a systemic immune response.

Immunogenic cell death describes the cellular death pathway triggered by the chronic exposure of DAMPs to the immune system. This immune-stimulatory form of apoptosis promotes an adaptive immune response to the dying cell. In oncology, this process appears to promote the immune recognition of malignant cells and anti-tumor immunity [142].

The electromagnetic interaction resulting from the electric heterogeneities in the tumor tissue offers a selection opportunity for malignant cells, causing stress, which allows for the manipulation of HSP production. Therefore, the applied electric field causes stress via both thermal and nonthermal processes.

The malignant cells have a higher concentration of membrane microdomains (rafts) than the nontumorigenic cells [82]. These rafts are selectively heated and reach a higher temperature than the TME [98,143]. The temperature increase of these nanoclusters is more rapid than the increase in the associated TME [105,144]. Some of these rafts act as stress sensors, operating as a trigger for the apoptotic cellular processes [4]. The cataphoretic forces generated by the modulated electric field induce lateral movements of electrically charged particles sensed by the rafts in the membrane. This results in the activation of the HSF1 and ultimately in the modulation of the actual HSP (mainly HSP70) levels. The heating creates optimal thermal conditions for the nonthermal molecular excitation of the transmembrane proteins in the lipid rafts, exciting the TRAIL death-receptor complexes and triggering the intrinsic apoptotic signals [109].

The initial peak of HSPs between 12 and 24 h post-treatment likely represents a coexistence of iHSPs and mHSPs. The second peak of HSPs seen several days after treatment with mEHT (Figure 6) is likely due to the extracellular expression of HSP70 proteins [101]. The intensive iHSP70 production characteristically follows all thermal shocks, independent of the heating technique [75], followed by the movement of iHSP70 to the cell membrane, forming mHSP70. The liberation of mHSP70 into the ECM appears only in the early stages of apoptosis in the studied preclinical models [106,107].

The gradual temperature increase applied in the step-up heating protocol of mEHT induces more HSPs in healthy cells than in the heavily stressed malignant cells [15]. The difference in the chaperone development results in a moderate increase in the expression of protective, antiapoptotic iHSP70 in the malignant cells compared to its drastic increase in the neighboring healthy cells [15]. This phenomenon provides an additional selective opportunity in the targeting process as the difference makes the malignant cells more vulnerable to stress than healthy cells. Moreover, step-up heating accounts for the blood wash-out time, maintaining the homeostatic equilibrium during the heating periods. Despite the development of the protective iHSP in malignant cells, thermo-resistance does not conflict with the administration of concurrent oncologic treatments as the natural physiological regulation boosts the blood circulation in the heated volume, which increases drug delivery in the case of chemotherapy, and increases oxygen perfusion necessary for the radio-sensitization of the tumor. During the heating-up period of mEHT treatments, the apoptosis rate is significantly higher than during the stable power output periods [113]. The applied step-up heating procedure could use this difference to improve the apoptotic processes [92]. As the temperature increases, the thermal effect could increase, and the excitation (nonthermal effect) may also increase due to the increased molecular excitation rates. During the periods of stable temperature, the thermal and nonthermal factors are constant, and the absorbed energy replaces the heat loss in the system.

In the pre-clinical studies, temperature measurements were taken during mEHT and classical heating techniques, such as water bath heating and capacitive heating. Most of the studies evaluated the samples from the different heating methods at the same temperature. However, Andocs et al. also compared mEHT heating at 38 degrees Celsius and 42 degrees Celsius to other heating methods at 42 degrees Celsius. This study revealed the nonthermal effects (field effects) as well as the thermal effects of mEHT. During mEHT, the thermal and nonthermal effects occur together, working synergistically [92,145]. The precise electromagnetic impedance tuning optimizes the synergy [146]. The two effects rely on the electromagnetic stimuli’s adherence to an intensity limit. A thermal load that is too high may destroy the excited receptor proteins, which could block the major pathways of the nonthermal effects.

Furthermore, high nonthermal doses can stop, or even decrease, the expression of iHSPs [147,148]. The overheating of tissues also cause a significant technical challenge. The forced absorption of high amounts of energy heats up the tissues non-selectively, producing isothermal conditions, marginalizing the selection conditions and overemphasizing the thermal component of the complex treatment effect.

In the case of high nonthermal doses, the strong apoptotic forces overcome the protection facility of the iHSPs, destroying the cellular integrity [106]. The iHSPs may translocate to the plasma membrane [51], forming mHSPs. The membrane expression of the major HSPs (HSP25, HSP60, HSP70, HSP90) has been observed [149], but their function was not readily apparent. They appear to either protect the cell [150] or participate in the immune stimuli [151,152], which act against the cell, resulting in mixed theories regarding the prognostic value of mHSPs on malignant cells [153,154]. The translocation process from iHSP to mHSP is also not yet completely understood. One proposal involves the “flip-flop” transition following the binding of iHSP to phosphatidylserine (PS) in tumor cells, facilitating the transport of HSP70 from inside the cell to the outer leaflet of the cell membrane [155]. This proposal aligns with the vibrational effect of the modulated electric field and the associated electro-osmotic process [156]. However, HSP expression on the cell surface alone does not provide enough signal for immune stimulation. The mHSPs must first form a complex tumor peptide to signal the apoptotic process [157]. Proteotoxic stresses, such as heat and chemical, pathological, and electromagnetic stresses, result in the formation of unfolded, aggregated, and ubiquitinated proteins. Heat shock proteins, such as iHSP70 and mHSP90, stabilize and refold these proteins, preventing their degradation [158]. The mHSP90 may, however, also trigger the DC activation signal required for a cell-to-cell interaction between the dying tumor cell and DCs [159].

**Figure 17:**
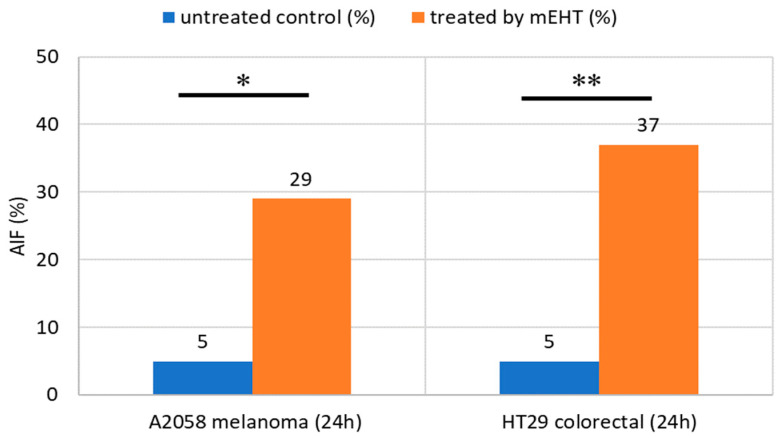
The enrichment of NK cells after 24 h of mEHT treatment in xenograft models for human melanoma and colorectal tumors [116]. * *p* < 0.05, ** *p* < 0.01.

The tumor-specific mHSP70 enables the recognition of the malignant cells by NK cells [55,160]. The mHSP70, found mainly in the cholesterol-rich microdomains of the cell membrane [161], activates the NK cells during an immune response [55,117]. The mEHT process supports the expression of mHSPs and promotes the NK-cell migration to the treated malignant cells (Figure 17) [111]. This extensive enrichment of NK cells in the tumor was measured in human melanoma (A2058) xenografts. The granzyme B expression increased such that 100% of the population stained positive in flow-cytometry measurements. The dead area of the tumor was measured by staining and fluorescent visualization in vivo. The dead area was also significantly increased following mEHT, and the cleaved Caspase3 was visualized with high intensity, indicating the pathway of the apoptotic cell death (Table 1).

The therapeutic goal in the case of mEHT is to both induce the local (treated tumor) and systemic (distant tumors and tumor cells) effect, and the local treatment could produce tumor-specific immune reactions which tackle the systemic targets. We propose using electromagnetically triggered apoptosis to target the local tumor to achieve this. While the local excitation triggers the increased expression of iHSPs, the electric field triggers the transition of iHSPs to the ECM and promotes apoptotic cell death. The overexpression of the iHSPs in the malignant cells is quickly exhausted, paving the way for apoptosis [106]. Additionally, the expressed HSP60 activates cCas3 [162], promoting caspase-dependent apoptosis.

**Figure 18:**
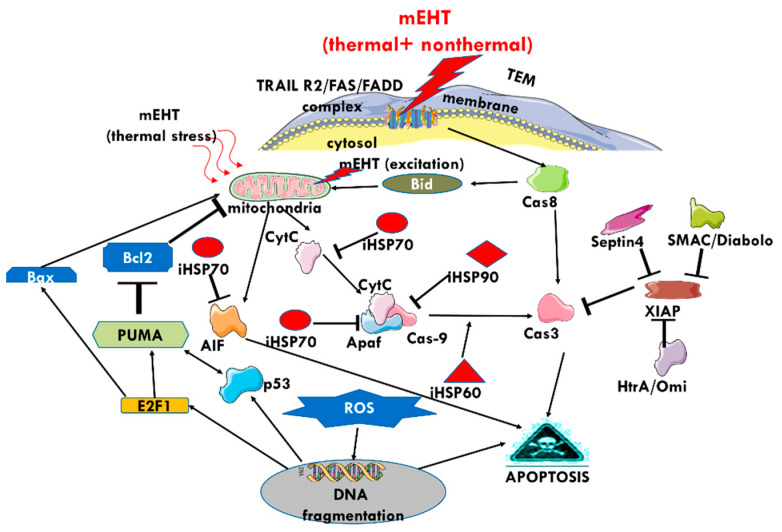
The multiple pathways involved in the apoptotic processes of cells after the thermal and nonthermal energy absorption of mEHT. The exhaustion of the protective mechanisms and the complex network of pathways ensures apoptosis as a final end-point.

The extrinsic excitation of rafts triggers the cooperation of $\mathrm{TRAIL}2\;\left(\mathrm{DR}5\right),\;\mathrm{FAS},\;\mathrm{and}\;\mathrm{FADD}$ [102]. The elevated expression of the collective group of molecules appears eight hours after treatment with mEHT [110]. The direct extrinsic path follows the Caspase-dependent pathway (Cas8→Cas3→apoptosis) [75]. The RF current actively changes various stress factors in the TME [163], potentially resulting in stress, which triggers the intrinsic pathway, starting from the mitochondria and following the Cas9→Cas3→apoptosis pathway [75]. The Caspase-independent pathway begins with the mitochondrial release of Cytochrome-C, the point of “no return” in the apoptotic process [75], resulting in the expression of apoptosis-inducing factors [102]. Measurements in preclinical studies have confirmed the increase in apoptotic cell death in mEHT-treated samples, resulting from the triggering of various signal pathways by the extrinsic excitation of death receptors in the selected membrane rafts [109]. Eventually, the numerous potential pathways triggered result in cell death via apoptosis (Figure 18). HSP70 impedes mitochondria-related apoptosis. The X-linked inhibitor of apoptosis protein (XIAP) inhibits both extrinsic and intrinsic signal pathways by obstructing cCas3. The mEHT treatment induces the expression of Septin4, impeding XIAP activity [164]. At eight hours post-treatment, SMAC/Diabolo and HtrA2/Omi mitochondrial regulatory proteins are also expressed [102], which also inhibit XIAP and support the caspase-dependent and -independent apoptotic pathways. The HSP60 promotes the (Cas9→cCas3) signal.

The set of DAMPs triggered by mEHT-induced apoptosis further paves the way toward promoting the immune targeting of systemic disease. DAMPs have variants of molecular sets expressed in response to different stimuli, and these variants are all hallmarks of various types of cell death [165]. The mEHT technique produces thermal and nonthermal mechanisms of electric fields synergistically promoting a specific set of DAMP molecules (Figure 16) as a result of the applied modulation. Multiple mechanisms relate to DAMP release [166]. The various DAMPs contain the HMGB1, eHSP70, and ATP, but numerous other molecules are released together, including some inflammatory molecules.

The key to eliciting systemic immunogenic effects against malignant lesions with mEHT is the promotion of the molecular sequences leading ICD [167]. The membrane rafts absorb the thermal and nonthermal energy, while the applied modulation orchestrates the spatiotemporal order of the exported DAMP-associated molecules to the TME, resulting in the desired ICD [101]. The ICD process releases a DAMP set of HMGB1, ATP, and eHSP70 in a time sequence, starting with membrane secretion of CRT. The mEHT-induced apoptosis delivers eHSPs together with other molecules to the TME, where the eHSP70 has a complex function [22] and acts as an “info signal” [12], which is pivotal in the orchestration of the immune activities.

The extensive heating in conventional homogeneous hyperthermia processes causes necrosis. The heat triggers the expression of iHSPs, which are mostly antiapoptotic. The necrotic cellular rupture also produces eHSP70 as the iHSP70 is released into the extracellular matrix [168,169]. However, the necrosis-induced eHSP does not offer a stable process to produce the desired antitumor immune promotion. Instead, in this instance, the eHSP in the ECM could work against further degeneration of the malignant cells, acting as a pro-tumor agent [170]; it may even regulate the DC capacity inducing immunosuppressive $T_{\mathrm{reg}}$ cells [171]. The $T_{\mathrm{reg}}$ inhibition presents another example of the opposing actions of the HSPs.

During hyperthermia, the arteries deliver blood, which acts as a cooling media. However, the distribution of the vasculature throughout the tumor is not even, resulting in hot spots in poorly perfused regions. There is a risk that the control of the eHSP action is lost in these overheated micro-regions. Furthermore, the relatively high temperature in these heated regions (>40 °C) inactivates the immune cells [172], whose active participation in the immunogenic processes is essential.

The mEHT method attempts to bypass the necrotic process, avoid inflammation, and suppress immunity [173]. The mEHT treatment promotes optimal DAMP production in a natural molecular cascade, including CRT expression on the cytoplasmic membrane. In sudden necrosis, the DAMP is released together with tumor-supportive inflammatory factors and mostly bypasses the membrane-secreted CRT.

Preclinical studies confirm that the DAMP-inducing apoptosis dominates in mEHT, and the necrotic cell destruction is relatively low [111,164]. Therefore, the mEHT method can avoid sudden inflammatory reactions and possible local toxicity associated with necrosis [174]. The more “gentle” apoptosis-associated cell death presents the genetic information from the malignant cells in a controlled manner using molecular signal pathways [101]. An advantage of the mEHT method is that the targeted malignant cells achieve a high enough temperature to initialize apoptotic signals [79,144], while the relatively low temperature (≤40 °C) in TME promotes the infiltration of the immune cells in the tumor without affecting their functional form.

Furthermore, the apoptosis-inducing stress from mEHT does not degrade the various DAMP molecules, which can deliver the necessary information and sensitizing actions for both the native and adaptive immune processes, offering a way to develop tumor-specific immune effects. The eHSP presents the “info signal” as the carrier [175] of tumor-specific antigens [13]. The peptides delivered by the released eHSP70 during apoptosis can be recognized by the innate and adaptive immune system [176,177] and, with the support of other molecular members of DAMP, can assist in priming the DCs for maturation [178].

Calreticulin acts as the “eat me” signal for phagocytosis [179,180], and its exposure is connected to the ER stress response [181]. It also plays a complex regulatory function in homeostasis [182,183] and carefully controls the intracellular influx of ${\mathrm{Ca}}^{2+}$ [184]. Calreticuli is the first to appear in the set of DAMP molecules. The CRT-controlled strong ${\mathrm{Ca}}^{2+}$ influx appears to significantly contribute to the electromagnetic stress-induced apoptosis that follows mEHT [105].

The HMGB1, another member of DAMP, represents a “danger signal” [185]. The HMGB1 is usually present in the nucleus; however, following appropriate stress conditions, it translocates to the cytoplasm and is released to the ECM during cell death. The non-oxidized HMGB1 participates in immune activation; however, it supports the inflammatory response in its oxidized form [186]. Furthermore, the oxidized HMGB1 participates in immune tolerance [187] and may boost the immune checkpoint molecule PD-L1 expression, limiting the anticancer immunity [188]. Therefore, the oxidation status of HMGB1 determines the role of HMGB1 in DAMP [189]. The mild thermal process favors conditions that do not support oxidization. The ATP released as a stress response in the apoptotic phase [190] is the “find me” signal [191], and mostly follows an autophagy-dependent pathway [192]. Again, due to the complexity of living conditions, ATP, as an energy source for dynamic changes, could support other functions by supplying energy.

The DAMP, therefore, also has a “double-edged sword” effect [193], acting with or against the tumor by causing apoptosis or promoting tumor progression [194], and the final outcome is the result of the complex effects and interactions of the molecular components of the DAMP [195].

**Figure 19:**
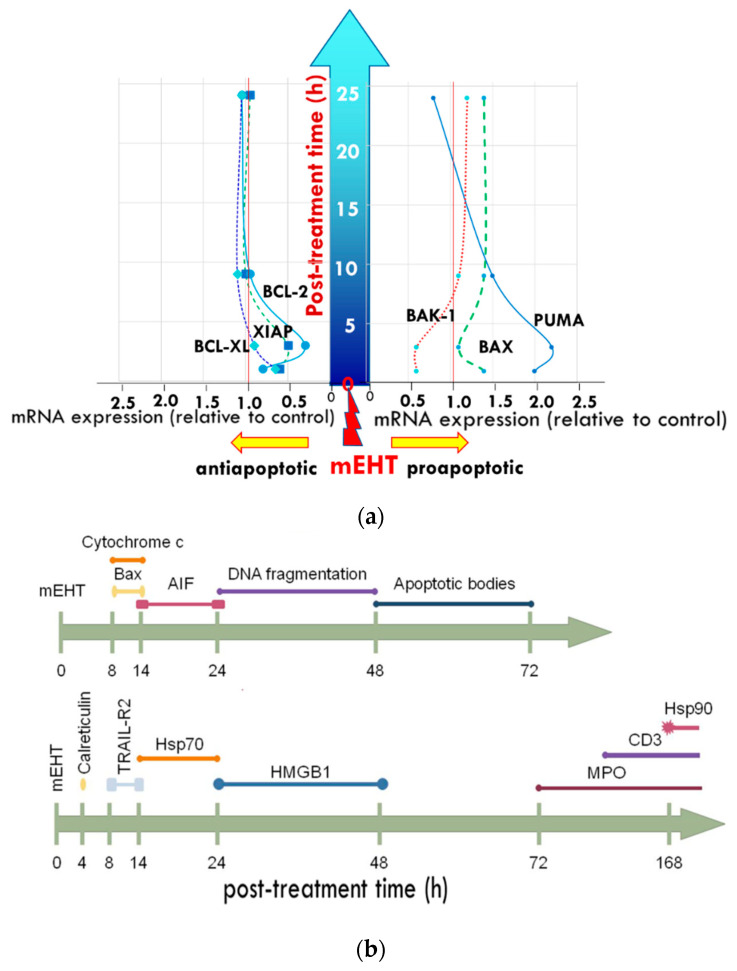
The time scale of the apoptotic processes: (**a**) development of pro-and anti-apoptotic proteins on the first day (24 h) post-treatment; (**b**) further developments with cellular reactions visible until the end of the apoptotic process (48 h post-treatment) and the immune reactions that follow three days after treatment.

The nonthermal effects of mEHT influence the homeostatic control using the time-fractal modulation [196]. The appropriate spatiotemporal order of DAMP molecules is crucial for their action. By applying the appropriate modulation, mEHT may trigger the appropriate timing of the appearance of various DAMP molecules in ECM [101,103] (Figure 19a,b).

In this proposal, the electric field effectively participates in limiting the malignant growth [197], harnessing the relatively low membrane potential of malignant cells [198], and creating clusters of cells [144]. The time-fractal modulated electric field in mEHT could potentially force autonomic malignant cells into collective groups [199], and the electromagnetic resonance may provide a reliable basis for molecular changes [87].

**Figure 20:**
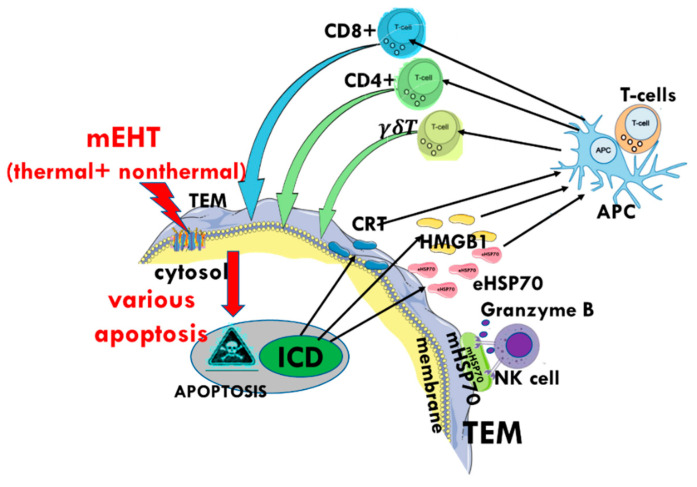
The apoptotic process exhausts the protective functions of iHSPs and secrets mHSP on the cytoplasmic membrane. The mHSP activates the NK-cells to attack the overstressed tumor cell, and the ICD process presents DAMP which could result in the maturation of the DCs, forming antigen-presenting cells (APCs). The APCs trigger the helper-T, killer-T, and γδT-cells, potentially resulting in a tumor-specific immune attack of the tumor cells all over the body (abscopal effect). The adaptive immune response is engaged, and the associated memory could protect the system from malignant relapses.

The tumor-specific immune activity of mEHT promotes the response of untreated distant metastases to the local irradiation of the primary tumor, known as the abscopal effect. The DAMPs with the delivered genetic information by eHSP70 activate DCs, generating antigen-presenting cells (APCs) [200]. This process induces the production of T-cells specific to the genetic information of the tumor cells supplied by the eHSP70 [122,123]. The eHSP70 provides the genetic information, with the cooperation of DAMP molecules, to form optimal conditions to maturate the DCs into APCs [201]. The APCs carry specific information about the malignant cell, and this material is used as a tumor antigen. The mature APC subsequently provides immunogenic information and produces tumor-specific killers ($\mathrm{CD}8^{+}$) and helper ($\mathrm{CD}4^{+}$) T-cells and activates antitumor T-cell immunity [202,203]. A noteworthy observation in cancer experiments is that mEHT treatment enhances the γδT−cell migration towards tumor cells even at temperatures as low as 38 °C [204]. The γδT−cells link the innate and adaptive immune systems [205], participating in the development of immune defenses. The APC induces numerous systemically available molecules (cytokines, chemokines), as well as NK, $\mathrm{CD}4^{+}$ and $\mathrm{CD}8^{+}$ cells and γδT-cells to form a complete tumor-specific immune arsenal which can be used against the malignant cells (Figure 20).

**Figure 21:**
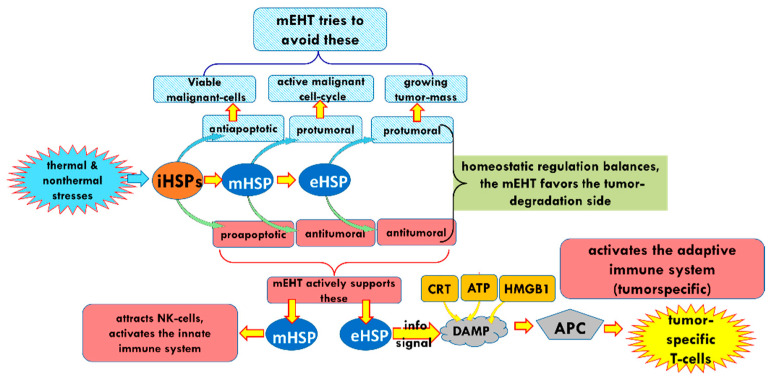
Summary of the role of HSP in the relevant processes.

The various HSPs have crucial roles in forming immune-specific antitumor processes all over the body, including targeting micro- and macro-metastases (Figure 21). The information delivered by the eHSP develops a tumor-specific adaptive immune response, which may even have the potential to prevent tumor seeding after a tumor re-challenge [113]. Following this, the eHSP, supported by the DAMP cellular collection, may promote antitumor immunity resulting in an anticancer vaccine effect [206].

## 7. Conclusions

Heat shock proteins have a complex “double-edged sword” effect, promoting and suppressing the malignant processes. Our goal is to apply an external stimulus, such as an electric field, to induce immune effects and tip the balance towards the side of tumor suppression, promoting the systemic immune recognition of tumor cells. Despite the plethora of literature on heat shock proteins, the precise roles and mechanisms of each HSP in adaptive and innate immunity are still not known. There is still much to learn about these molecules about the precise effects of mEHT and electric fields on the immune modulation of malignant cells. Following a review of the literature, we have proposed a potential mechanism for the outcomes seen in the preclinical and clinical data on mEHT, in which HSPs play a crucial role. The electric field’s synergistic action of the thermal and nonthermal effects selectively heats the tumor cells and targets its membrane components [91]. The nonthermal processes following an applied modulated field dominate [114] and effectively excite the voltage-related cellular receptors and channels on the plasma membrane, resulting in a significant reduction in the proliferation and clonogenicity of the malignant cells [76]. The application of mEHT can promote the antitumor HSP activity, and in situ it stimulates the tumor-specific immune effects, which act locally and may also play a role in the systemic management of disseminated cells and metastatic lesions. Enriching of mHSP70 activates the innate (NK-cell) immune attack on the selected malignant cells. The ICD-induced DAMP starts preparing the adaptive immune reactions by forming APCs. The spatiotemporal arrangement of DAMP complexly orchestrates the eHSP70 (“info”), CRT “eat-me”, HMGB1 “danger,” and ATP “find me” signals. The subsequent maturation of the DCs into APCs creates cytotoxic T-cells and triggers tumor-specific immune processes with a solid potential to act systemically. Therefore, modulated electro-hyperthermia is a potential tool for the manipulation of HSPs to achieve the goal of immunogenic recognition and targeting of the tumor, resulting in the local and systemic control of the disease.

## Figures and Tables

**Table 1:** NK cell activity and mEHT (** means *p* < 0.001 and *** means *p* < 0.0005).

Tumor (A2058 Melanoma (24 h))	Dead Area (%)			FI Signal Intensity			cCas3 (%)		
	mEHT + Primary NK	mEHT + NK92MI	mEHT Alone	mEHT + Primary NK	mEHT + NK92MI	mEHT Alone	mEHT + Primary NK	mEHT + NK92MI	mEHT Alone
Untreated control (%)	5	9	7	0.9	0.8	0.9	5	6	8.5
Treated by mEHT (%)	63	90	60	1.35	1.39	1.26	26	33	19
Significance		***			**	***	**	***	
